# Integrating dorsolateral prefrontal cortex multi-omics and GWAS summary data reveals genetic etiology of Parkinson’s disease

**DOI:** 10.21203/rs.3.rs-9622597/v1

**Published:** 2026-05-28

**Authors:** Qiang Liu, Shinya Tasaki, David A. Bennett, Nicholas T. Seyfried, Philip L. De Jager, Vilas Menon, Aron S. Buchman, Jingjing Yang

**Affiliations:** 1. Center for Computational and Quantitative Genetics, Department of Human Genetics, Emory University School of Medicine, Atlanta, GA, 30322, USA; 2. Department of Biostatistics and Bioinformatics, Emory University School of Public Health, Atlanta, GA, 30322, USA; 3. Rush Alzheimer’s Disease Center, Rush University Medical Center, Chicago, IL, 60612, USA; 4. Department of Biochemistry, Emory University School of Medicine, Atlanta, GA, 30322, USA; 5. Center for Translational and Computational Neuroimmunology, Department of Neurology and Taub Institute for Research on Alzheimer’s Disease and the Aging Brain, Columbia University Irving Medical Center, New York, NY10032

## Abstract

To better illustrate the genetic etiology of Parkinson’s disease (PD), we integrated xQTL weights derived from bulk RNA-seq (n=931), single-nucleus RNA-seq (n=415), and bulk proteomics (n=716) data of dorsolateral prefrontal cortex (DLPFC) with the largest available GWAS summary data of PD. Through integrative Omnibus TWAS and PWAS analyses, we detected risk genes whose genetic effects are mediated through bulk or cell-type-aware gene expression, or bulk protein abundances in DLPFC. We detected 39 significant risk genes by bulk TWAS, 66 by cell-type-aware TWAS across six brain cell types, and 17 by bulk PWAS. Importantly, 57.9% bulk and 62.5% cell-type-aware independent TWAS risk genes are replicated by bulk PWAS. Protein–protein interaction analyses reveal strong connectivity of our detected risk genes with known PD risk genes such as *MAPT*, *SNCA*, and *LRRC37A*. Our detected TWAS and PWAS risk genes are shown enriched in apoptosis signaling and T-cell activation pathways.

## Introduction

Parkinson’s disease (PD) is the second most prevalent neurodegenerative disorder worldwide, and its incidence is rising in step with global population aging^1^. Recent large-scale genome-wide association studies (GWASs) have uncovered ~90 independent PD risk loci^2,3^. However, the underlying genetic etiologies of these identified GWAS risk loci are still largely unknown. A recently developed transcriptome-wide association study (TWAS) and proteome-wide association study (PWAS) approach integrates other molecular quantitative traits such as gene expression or protein abundance with GWAS summary data, with the goal of illustrating molecular mechanisms of identified PD risk genes^4–7^.

TWAS/PWAS tools often first estimate molecular quantitative trait loci (xQTL) weights using a reference panel that contain data of both genetic and the molecular trait, and then conduct gene-based association tests by integrating these xQTL weights and large-scale GWAS summary data. A recent study also shows that the TWAS/PWAS approach is equivalent to the probability Mendelian Randomization analysis, which can test if the causal genetic effect of the target gene is mediated through the considered molecular trait^8^.

Recent TWASs of PD^9–12^ have nominated dozens of susceptibility genes, yet these studies relied on bulk RNA-seq reference panels and used a single statistical model to estimate xQTL weights, limiting the discovery power for exploring cell-type-aware causal genes and genes whose genetic effects are mediated through protein abundances. These analyses are critical to advance integrative single-nucleus multi-omics studies that have reported the potential importance of astrocyte, microglia, and oligodendrocyte cells in the disease mechanisms underlying PD^13–15^. For example, PD-associated variants displayed cell-type-specific enhancer enrichment in mouse, with strong signals in microglia and oligodendroglia^14^. Single-nucleus RNA-seq (snRNA-seq) analysis of mid-brain and cortex in PD cases has revealed pronounced, cell-type-specific transcriptional dysregulation, particularly within microglia and astrocytes, and shown significant enrichment of PD heritability in microglial open-chromatin regions^13,16^. Another snRNA-seq analysis of cis-regulatory elements in substantia nigra in PD cases has uncovered new potential biomarkers, delineating disease-perturbed pathways, and highlighting the pathogenic roles of astrocytes and microglia cells^15^. Together, these findings highlight the importance of integrating large cohorts of bulk RNA-seq, snRNA-seq, and bulk proteomics data, with GWAS summary data for elucidating the varied genomic mechanisms underlying PD.

To bridge these knowledge gaps about the genetic etiology of PD, this study integrated the expression quantitative trait loci (eQTL) weights derived from large cohorts of older adults with bulk RNA-seq (n=931) and snRNA-seq (n=415) data of dorsolateral prefrontal cortex (DLPFC) tissue^17,18^ with PD GWAS summary data^2^ (n=~1.45M), for mapping risk genes (i.e., TWAS) of PD. Cell-type-aware TWAS was conducted using the eQTL weights derived within six major cortical cell types –– astrocytes, microglia, excitatory neurons, inhibitory neurons, oligodendrocytes, and oligodendrocyte precursor cells (Opcs). Additional analyses integrated protein quantitative trait loci (pQTL) weights derived from a large cohort of tandem mass tag (TMT) proteomics data (n=716) of DLPFC tissue^18^ with GWAS summary data of PD^2^ (i.e., PWAS).

Three sets of xQTL weights^18^ were estimated by three complementary methods: nonparametric Bayesian Dirichlet Process Regression (TIGAR/DPR)^19^, penalized regression with Elastic-Net penalty (PrediXcan/EN)^20^, and the best-performing model selected by FUSION based on 5-fold cross-validation (CV) *R*^2^ (FUSION/BestModel)^21^. We utilized our recently developed Omnibus TWAS/PWAS (xWAS-O) method^22^ to aggregate TWAS/PWAS p-values based on these three sets of xQTL weights per test gene, for detecting significant risk genes of PD with improved power than considering only one set of xQTL weights^23^. We identified 39 significant risk genes by bulk TWAS, 66 by cell-type-aware TWAS, and 17 by PWAS, resulting in 19, 32, and 11 independent risk genes by bulk TWAS, cell-type-aware TWAS, and bulk PWAS, respectively.

By comparing significant risk genes detected by bulk TWAS and cell-type-aware TWAS with the ones detected by bulk PWAS, we examined if the test eQTL of significant TWAS risk genes overlapped with the test pQTL of significant PWAS risk genes. If overlapped, the genetic effects of these TWAS risk genes are potentially mediated through their gene expression and the corresponding protein abundances, which can be viewed as a validation of identified TWAS risk genes. Importantly, 11 out of 19 (57.9%) independent bulk TWAS risk genes, and 20 of 32 (62.5%) independent cell-type-aware TWAS risk genes, were replicated by bulk PWAS.

To identify possible causal variants for identified significant TWAS and PWAS risk genes, we a) implemented fine-mapping using the Genomic Integration Fine-mapping Tool^24^ (GIFT); b) conducted protein–protein interaction (PPI) analyses by STRING^25^; and c) explored enriched gene pathways by pathDIP^26^. PPI network analyses demonstrated that cell-type-aware TWAS risk genes were interconnected with known PD risk genes such as *MAPT*^27^, *SNCA*^28^, and *LRRC37A*^29^, and enriched in apoptosis signaling and T-cell activation pathways. TWAS and PWAS risk genes detected in this study were both found enriched in cholecystokinin receptor (CCKR) signing map and T-cell activation pathways.

## Results

### Identifying TWAS-O and PWAS-O risk genes of PD by xWAS-O framework

In this study, we utilized our previously developed xWAS-O framework^22^ to integrate large-scale GWAS summary data of PD^2^ (n=~1.45M) with three sets of estimated xQTL weights of DLPFC^17,18^ by TIGAR/DPR^19^, PrediXcan/EN^20^, and the FUSION/BestModel^21^ based on complementary statistical models with (see Methods). Bulk RNA-seq, snRNA-seq, and bulk TMT proteomics data that were profiled from the DLPFC samples of the ROS/MAP^30–33^ cohorts, along with their genetic data, were used to estimate these xQTL weights for each quantitative molecular trait^17,18^. The GWAS summary data (n=~1.45M) of PD were generated by meta-analysis of European cohorts^2^, including a total of 37,688 clinically diagnosed PD cases, 18,618 proxy PD cases, and approximately 1.4 million controls.

The genetically regulated molecular traits that have 5-fold cross-validation (CV) $R^{2}>0.5\%$ for any one of these three statistical methods in the reference data were tested against the PD phenotype in the PD GWAS summary data^2^. Using the Aggregated Cauchy Association Test (ACAT) method^34^, xWAS-O^19^ combines TWAS/PWAS p-values based on each set of the xQTL weights per gene to obtain xWAS-O p-values. In total, we tested 13,397 bulk gene expression traits, about 13,000 cell-type-aware gene expression traits for each of the six cortical cell types (astrocytes, microglia, excitatory neurons, inhibitory neurons, oligodendrocytes, and Opcs), and 4,710 protein abundance traits. Significant TWAS-O genes were defined as those with TWAS-O p-values < 2.5 × 10^−6^ (genome-wide significance level for gene-based association studies). Because only a subset of protein coding genes were tested by PWAS-O (4,710), significant PWAS-O genes were defined as those with false discovery rate (FDR) q-values < 0.05 after genomic control factor adjustment^35^ and multiple test correction by Benjamin-Hochberg procedure^36^.

Using the Gene-based Integrative Fine-mapping through conditional TWAS method (GIFT)^24^ (see Methods), we fine-mapped independent TWAS-O/PWAS-O signals in nearby genomic regions containing multiple significant risk genes for calibrated the inflated false positive rate. Briefly, for each genomic region defined as ±1 Mb around a top significant gene, GIFT jointly modeled the genetically regulated molecular traits of all genes in the region while accounting for linkage disequilibrium (LD) in the test genome region and correlations among the considered molecular traits. TWAS-O risk genes that either have fine-mapped GIFT p-values <0.05 or contain no overlapped test cis-SNPs with other nearby significant TWAS-O genes are considered as independent signals.

### Bulk TWAS-O findings

Quantile-Quantile plots of bulk TWAS results based on three statistical methods and by TWAS-O are presented in **Fig. S1.** As expected, inflated false positive rates are observed in all TWAS results. This arises from several contributing factors: inflated GWAS summary statistics due to LD, overlapping test regions among nearby genes, and correlations among the molecular traits of neighboring genes. We identified 39 TWAS-O significant risk genes of PD, 17 of which were reported as associated with PD-related traits in GWAS Catalog^37^ (Table 1; **Supplementary Data 1**).

**Figure 1.**
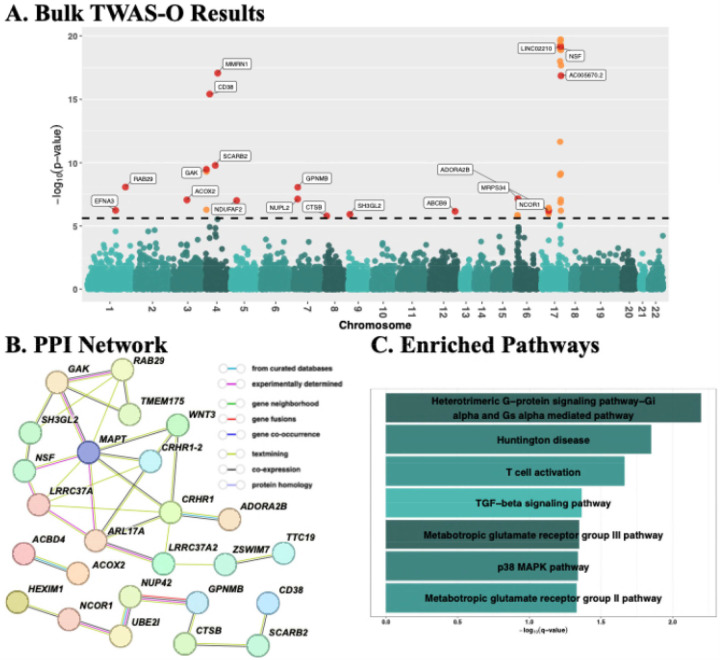
Bulk TWAS-O results of PD. A.) Manhattan plot of -log10(bulk TWAS-O p-values): Transcriptome-wide significance threshold -log10(2.5E-06) was plotted as the dashed horizontal line. Independent significant TWAS-O risk genes are colored in red and labelled. Other non-independent ones are colored in orange. B.) PPI network of significant bulk TWAS-O risk genes: Edges in the PPI network plot represent PPI links, with different colors representing different sources of connection evidence as shown in the legend. C.) Pathways significantly enriched with our bulk TWAS-O findings are plotted, with the -log10(enrichment FDRs) in the x-axis.

By GIFT, from the total of 39 TWAS-O significant risk genes, we fine-mapped 19 independent signals (**Fig. S9**) that were colored in red and labeled in the Manhattan plot Fig. 1A. Among those 19 independent risk genes, 7 are not reported as associated with PD in GWAS Catalog^37^ (bold in Table 1) –– *ACOX2* encoding a peroxisomal enzyme involved in the β-oxidation of branched-chain fatty acids^38^; *NUPL2* identified as part of protein ubiquitination and degradation pathways that were shown implicated in PD pathology such as α-synuclein aggregation and impaired protein clearance^11^; *ABCB9* encoding ATP binding Cassette Subfamily B member 9 whose methylation was associated with PD^39^; *MRPS34* that was associated with mitochondrial translation and suspected causing Leigh syndrome, might also promote neuronal vulnerability in PD^40^; *AC005670.2* and *ADORA2B* were associated with PD in previous studies^41,42^; *NCOR1* coding a nuclear receptor corepressor, whose gene expression was shown significantly upregulated in the substantia nigra of PD patients^43^, implicating its involvement in PD pathogenesis^41^. As described here, several of these bulk TWAS-O risk genes are not reported in GWAS Catalog but are shown with PD related biological functions in previous studies.

### Cell-type-aware TWAS-O findings

**Figure 2.**
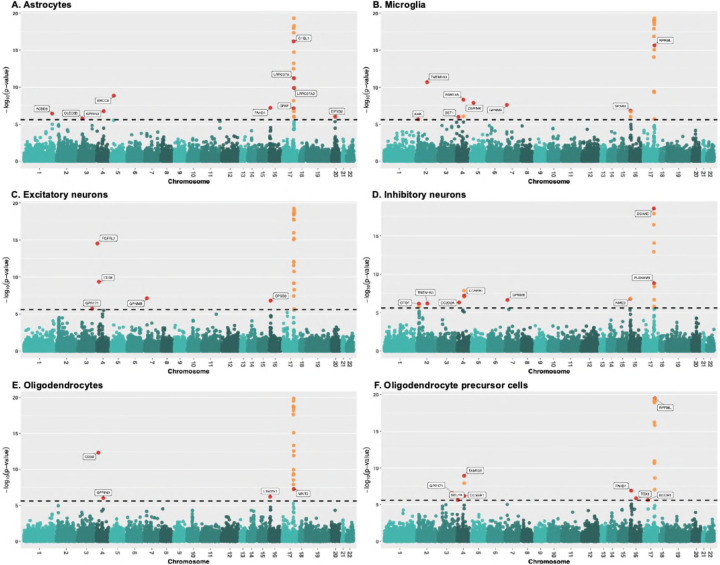
Cell-type-aware TWAS-O results of PD in astrocytes, microglia, excitatory neurons, inhibitory neurons, oligodendrocytes, and Opcs. Transcriptome-wide significant threshold -log10(2.5E-06) was plotted as the dashed horizontal line. Independent significant genes are colored in red and labelled, including the ones detected by GIFT and the ones having no overlapped test regions with other significant cell-type-aware TWAS-O risk genes. Other non-independent cell-type-aware TWAS-O risk genes are colored in orange.

Quantile-Quantile plots of the cell-type-aware TWAS results are shown in **Figs. S2-S7**. The Manhattan plots of cell-type-aware TWAS-O results are shown in Fig. 2. Independent cell-type-aware TWAS-O risk genes are listed in Table 2. We compared cell-type-aware TWAS-O to bulk TWAS-O findings. A cell-type-aware TWAS-O risk gene was considered an overlapping finding in bulk if it was also detected by bulk TWAS-O, or if its tested eQTLs overlapped with that of a significant bulk TWAS-O gene. Cell-type-aware TWAS-O risk genes are considered novel findings if they are not reported in GWAS Catalog or overlapped with any bulk TWAS-O risk gene (bolded in Table 2).

**Figure 3.**
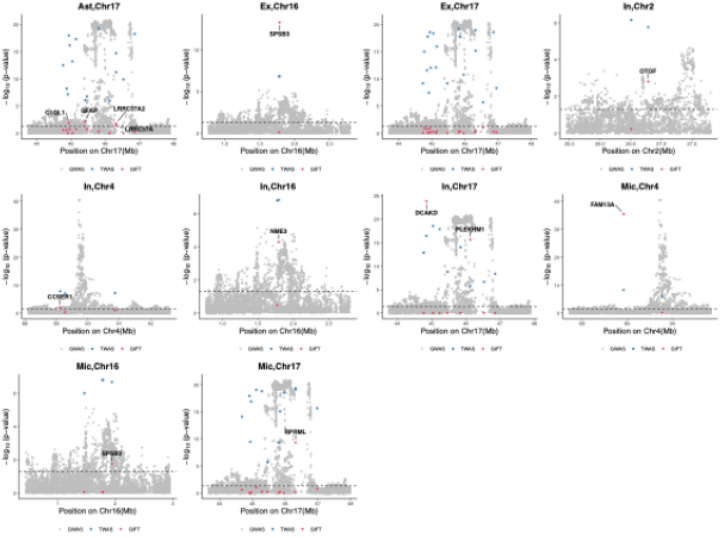
Fine-mapped cell-type-aware TWAS-O risk genes of PD by GIFT in astrocytes, microglia, excitatory neurons, and inhibitory neurons. The -log10(p-values) were plotted on the y-axis, and -log10(0.05) was plotted as the dashed horizontal line. Gray dots: SNPs by GWAS; Blue squares: genes identified by TWAS; Red diamond: results by GIFT. Causal significant genes are labeled.

For astrocytes, we identified 21 significant TWAS-O risk genes (Fig. 2A; **Supplementary Data 2**), including 5 that were previously linked to PD clinical or pathological traits in GWAS Catalog^37^. Using GIFT, we identified 10 independent signals (Table 2; Fig. 3) including 3 novel findings –– *ACBD3* encoding a Golgi-resident acyl-CoA-binding domain-containing protein that participates in lipid trafficking, apoptosis, and neuronal differentiation^44^; *CLEC3B* encoding tetranectin, a C-type lectin that promotes plasminogen activation, normally presents in serum, CSF, and extracellular matrix. Particularly, CSF tetranectin is shown reduced in PD patients^45^. The genetic or siRNA depletion in *CLEC3B* accelerates p53/Bax-mediated dopaminergic neurodegeneration and motor impairment, while the over-expression of *CLEC3B* is neuroprotective, supporting *CLEC3B* as both a potential biomarker and therapeutic target for PD^45^. *EIF2S2* encodes eukaryotic initiation factor subunit 2 (EIF2) and EIF2 signaling is observed in dopaminergic neurons from PD patients^46^.

For microglia, we identified 24 significant cell-type-aware TWAS-O risk genes (Fig. 2B; **Supplementary Data 3**), including 8 that were previously reported to be associated with PD-related traits in the GWAS Catalog^37^. Using GIFT, we identified 8 independent signals (Table 2; Fig. 3), including 1 novel finding - *KHK* encoding ketohexokinase, the rate-limiting enzyme that converts fructose to fructose-1-phosphate. A previous study revealed that KHK-dependent fructose metabolism in microglia drives NOX4-mediated oxidative stress, a pathway pertinent to dopaminergic neurodegeneration^47^.

In neurons, we identified 25 significant cell-type-aware TWAS-O risk genes in excitatory neurons (**Supplementary Data 4**) and 19 in inhibitory neurons (**Supplementary Data 5**), including 6 and 9 that were previously associated with PD-related traits in GWAS Catalog^37^. We fine-mapped 5 and 8 independent signals by GIFT, respectively (Table 2; Fig. 3). One novel independent gene was identified in excitatory neurons - *GPR171* encoding an orphan class-A GPCR for the neuropeptide BigLEN. The blockade of this receptor was shown to unleash antitumor T-cell activity^48^, and the BigLEN precursor proSAAS protects dopaminergic neurons and limits α-synuclein spread in rodent models^49^. One novel independent gene was identified in inhibitory neurons –– *OTOF* encoding otoferlin, a multi-C2-domain Ca^2+^ sensor essential for ribbon-synapse vesicle fusion in auditory inner hair cells. Importantly, single-cell profiling of MPTP-parkinsonism macaques identified *FOXP2*^+^ *OTOF*^+^ inhibitory neurons within the nigrostriatal system^50^.

In oligodendrocytes and Opcs, we respectively identified 21 and 23 significant cell-type-aware TWAS-O risk genes (**Supplementary Data 6–7**), including 9 and 6 that were previously associated with PD-related traits in GWAS Catalog^37^. We fine-mapped 4 and 8 independent signals by GIFT, respectively (Table 2; **Fig. S10**). We identified no novel independent risk genes in oligodendrocytes, and two novel independent risk genes in Opcs –– *GPR171*^48,49^ that was also detected in excitatory neurons as described above; *TOX3* encoding a protein with a high-mobility-group (HMG) box that can bend and unwind DNA, thereby modulating chromatin architecture^51^. A previous case-control analysis of SNPs at the *TOX3* locus revealed that homozygous carriage of rs3104767 nominally increases PD risk^52^.

### Bulk PWAS-O findings

Quantile-Quantile plots of PWAS results by three tools and PWAS-O are presented in **Fig. S8**. We identified 17 PWAS-O significant risk genes of PD with q-values<0.05, including 4 reported as associated with PD-related traits in GWAS Catalog (**Supplementary Data 8**). By GIFT, we fine-mapped 11 independent PWAS signals (**Fig. S11**). We detected 3 novel findings that were not reported as associated with PD in GWAS Catalog, nor detected by our bulk TWAS-O analyses (Table 3) – *SNX17* whose depletion was shown decreasing steady-state levels of amyloid precursor protein with a concomitant increase in Aβ generation^53^; *EXOSC7* encoding Exosome Component 7; *VPS26B*, a core retromer subunit, whose upregulation protects neuronal function, suggesting its neuroprotective role in PD^54^.

Out of 17 unique PWAS-O risk genes, 5 (29.4%) were detected by bulk TWAS-O and 6 (35.3%) were detected by cell-type-aware TWAS-O, as shown by the Venn diagram in **Fig. S12**. We further calculated the pairwise Jaccard Similarity Indexes^55^ using the same lists of genes by bulk TWAS-O, cell-type-aware TWAS-O, and PWAS-O, which quantifies the overlap proportion of significant risk genes (**Fig. S12**). Here, only the same genes identified in both analyses are considered as overlapping. We found that the pair-wise cell-type-aware TWAS-O findings have 10%~36% overlaps, with top overlaps 36% between excitatory neurons and microglia, 35% between excitatory neurons and astrocytes, and 35% between oligodendrocytes and astrocytes. Further, we found that cell-type-aware TWAS-O findings have 4%~15% overlaps with bulk TWAS-O findings, with top overlaps found in excitatory neurons (14%) and oligodendrocytes (15%). We found that cell-type-aware TWAS-O findings have 3%~14% overlaps with PWAS-O findings, with top overlaps also found in excitatory neurons (14%), and oligodendrocytes (9%). These results show important roles of excitatory neurons and oligodendrocytes.

### Validating cell-type-aware TWAS-O findings

We further use PWAS-O findings to validate our detected independent cell-type-aware TWAS-O risk genes. For each of the independent cell-type-aware TWAS-O risk genes, if its test gene region is overlapped with the test regions of any significant PWAS-O genes, it is considered as validated in proteomics (Table 4).

In astrocytes, we identified 6 of 10 (60%) independent TWAS-O signals overlapping with PWAS-O findings, 5 of which (*CLEC3B*, *C1QL1*, *GFAP*, *LRRC37A*, and *LRRC37A2*) were specific for astrocytes. *C1QL1* encodes a secreted C1q/TNF-related protein that engages C1ql1-Bai3 signaling that mediates CF synaptogenesis in mature Purkinje cells^56^. Its central role in cerebellar synaptic homeostasis and the motor-coordination changes observed in C1ql1-deficient mice suggest it may modulate motor impairment derived from neuronal degeneration in PD models^57^. *GFAP* encodes one of the major intermediate filament proteins of mature astrocytes. In PD patients, plasma *GFAP* is elevated and may have utility for subtyping PD traits and their conversion to PD over time^58^. *LRRC37A/2* encodes a membrane-associated protein involved in cellular migration, chemotaxis and astroglia inflammation, which has been reported to be co-localized with Lewy bodies in PD brain tissue^29^.

In microglia, 5 out of 8 (63%) independent TWAS-O signals overlapped with PWAS-O findings (Table 4) but only 2 of 5 genes were validated in microglia (*KHK*^47^ and *BST1*). Bone marrow stromal cell antigen-1 (*BST1*), is a glycosylphosphatidylinositol-anchored surface protein expressed by stromal cells that promotes pre-B-cell proliferation^59^ and rs4698412 SNP in *BST1* is associated with disease progression in PD^60^.

In excitatory neurons, 3 of 8 (38%) independent TWAS-O signals overlapped with PWASO findings; in inhibitory neurons, 6 of 8 (75%) independent TWAS-O signals overlapped with PWAS-O (Table 4). None of genes in excitatory neurons validated in proteomics are cell-type-specific, and 5 of the validated genes in inhibitory neurons (*OTOF*^50^, *CC2D2A*, *NME3*, *DCAKD*, and *PLEKHM1*) are cell-type-specific. In particular, coiled-coil and C2 domain-containing protein 2A, encoded by *CC2D2A*, forms a scaffold protein at the ciliary transition zone^61^. SNPs near *BST1*, a TWAS-O risk gene in microglia that was validated in proteomics, are linked to altered *CC2D2A* expression in astrocytes of PD patients, suggesting its potential role in PD^62^. A previous study^63^ showed that *NME3* encodes a nucleoside diphosphate kinase, whose reduced expression in the substantia nigra is associated with the protection against dopaminergic neurodegeneration in a 1-methyl-4-phenyl-1,2,3,6-tetrahydropyridine (MPTP)-induced PD mouse models. *DCAKD* enables dephospho-CoA kinase activity, which has been highlighted in a previous TWAS of PD^64^. Pleckstrin homology domain–containing family M member 1 (*PLEKHM1*) is a lysosomal adaptor supporting endosome- and autophagosome-lysosome fusion^65^, whose dysregulated expression is causally linked to PD risk, implicating defective protein clearance in PD pathogenesis^66^.

In oligodendrocytes, 3 of 4 (75%) independent TWAS-O signals overlapped with PWAS-O findings; in Opcs, 3 of 8 (38%) independent TWAS-O signals overlapped with PWAS-O findings (Table 4). We found that 2 (*C16orf91*, *WNT3*) and 1 (*NCOR1*^41,43^) of these validated genes in oligodendrocytes and Opcs were cell-type-specific, respectively. Interestingly, *C16orf91* is involved in mitochondrial respiratory chain complex assembly^67^. Wnt family member 3 (*WNT3*) encodes a canonical Wnt ligand that engages the β-catenin pathway. Astrocyte-secreted Wnt3 in the dentate gyrus has been shown to accelerate adult hippocampal neurogenesis, and recombinant Wnt3 is routinely incorporated into protocols that push human embryonic stem cells toward mid-brain dopaminergic lineages^68^.

### Replicating TWAS-O/PWAS-O risk genes in ROS/MAP cohort

To further assess whether significant PD risk genes detected by the above TWAS-O/PWAS-O analyses are also related to motor impairment and PD neuropathologic traits within ROS/MAP, we performed follow-up replication analyses using individual-level phenotypes of global parkinsonian score (n=1,187) and pathologic PD^69,70^ (n=1,194), and WGS genotype data (i.e., GWAS data). Global parkinsonism score is a continuous trait for assessing four parkinsonian signs (parkinsonian gait, rigidity, bradykinesia, and tremor) using 26 items from a modified Unified Parkinson’s Disease Rating Scale^71,72^. Pathologic PD is a binary trait defined here as the presence of either nigral neuronal loss or Lewy body pathology (see detailed descriptions in Methods). Covariates age at death, sex, study indicator (ROS or MAP), and the top two genotype principal components were adjusted for in the analyses. Given a much smaller sample size in the individual ROS/MAP genotype and phenotype data (compared to the PD GWAS summary data^2^), we consider a gene is related to the tested trait and replicated if its TWAS-O/PWAS-O p-value < 0.05 using individual ROS/MAP GWAS data.

A total of 8 cell-type-aware TWAS-O risk genes and 1 bulk TWAS-O risk gene were found related to the global parkinsonian score (**Supplementary Data 9**), including *OTOF* in bulk tissue, *GPRIN3* and *TSR3* in astrocyte, *SNCA* in excitatory neurons, *CTSB* and *NELFA* in inhibitory neurons, *CLEC3B* and *CCDC43* in microglia, and *HEXIM2* in both excitatory neurons and microglia. Among these, *SNCA* is notable given its established role in α-synuclein aggregation and PD pathobiology^28^, suggesting that genetically regulated expression signals identified by TWAS may also relate to variation in motor impairment severity.

A total of 13 cell-type-aware TWAS-O risk genes, 4 bulk TWAS-O risk genes, and 7 bulk PWAS-O risk genes were found related to the pathologic PD, including several genes highlighted in the above TWAS-O and PWAS-O analyses using PD GWAS summary data: *OTOF*, *CLEC3B*, *ACOX2*, *BST1*, *KHK*, *ACBD3*, *VPS26B*, and *ADORA2B* (**Supplementary Data 9**). Notably, *OTOF* showed significant association with pathologic PD across multiple molecular contexts - bulk tissue, excitatory neurons, inhibitory neurons, and OPCs; *CLEC3B, ACOX2*, and *BST1* showed strong associations in both excitatory neurons and microglia; glial-lineage signals including *BST1*, *IDUA*, and *CD38* were associated with pathologic PD in microglia, oligodendrocytes, or Opcs.

### PPI network and enrichment analyses

**Figure 4.**
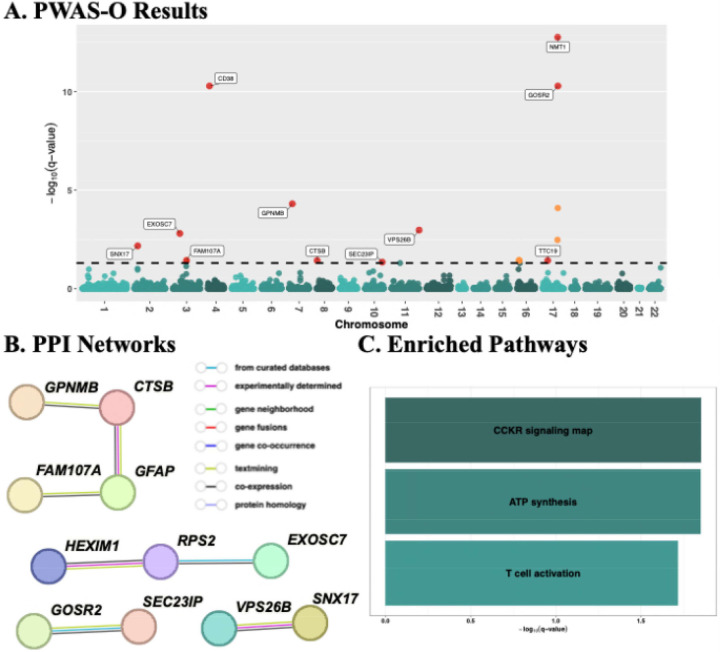
Bulk PWAS-O results of PD. A.) Manhattan plot of -log10(bulk PWAS-O q-values): Significance threshold -log10(0.05) was plotted as the dashed horizontal line. Independent significant PWAS-O risk genes are colored in red and labelled. Other non-independent ones are colored in orange. B.) PPI network of significant PWAS-O risk genes: Edges in the PPI network plot represent PPI links, with different colors representing different sources of connection evidence as shown in the legend. C.) Pathways significantly enriched with PWAS-O findings are plotted, with the -log10(enrichment FDRs) in the x-axis.

**Figure 5.**
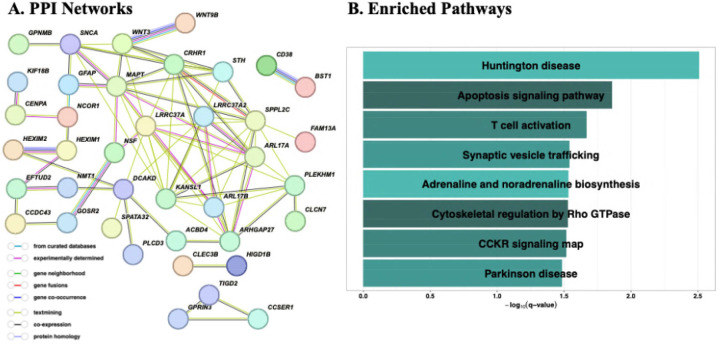
PPI network and pathway enrichment analyses results with cell-type-aware TWAS-O risk genes of PD in six major brain cell types. A.) Edges represent PPI links, with different colors representing different sources of connection evidence. TWAS risk genes not connected in the network are not included in the network plot. B.) Enrichment pathways significantly enriched with cell-type-aware TWAS-O risk genes of PD, with the -log10(enrichment FDRs) in the x-axis.

Using the STRING webtool^73^ (Methods), we conducted PPI network analyses using the lists of bulk TWAS-O risk genes, PWAS-O risk genes, and combined cell-type-aware TWAS-O risk genes of all six brain cell types. The edges of the PPI network represent PPI links and were colored according to different data sources as shown in the color legend (Fig. 1B, Fig. 4B, Fig. 5A). In addition, pathways significantly enriched with our bulk TWAS-O, PWAS-O, and cell-type-aware TWAS-O findings were found by the pathDIP tool^26^ (Fig. 1C; Fig. 4C; Fig. 5B; **Supplementary Data 10**).

A total of 25 of 39 (64.1%) bulk TWAS-O risk genes were interconnected in the PPI network plot (Fig. 1B). Notable genes include *MAPT* and *CRHR1*, which have been implicated in PD pathology. *MAPT*, which encodes the microtubule-associated protein tau, carries two major inversion haplotypes on chromosome 17. The common H1 haplotype has been reported to significantly over-represent in PD overall and conferred roughly a two-fold increase in risk for the non-tremor-dominant (NTD) motor subtype^27^. *CRHR1*, which encodes G-protein coupled receptor that binds to neuropeptides of the corticotropin-releasing hormone family, is shown associated with decreased risk of PD^74^.

Pathway enrichment analyses with bulk TWAS-O risk genes revealed PD relevant significant pathways (Fig. 1C). Specifically, the *Heterotrimeric G-protein signaling pathway-Gi alpha and Gs alpha mediated pathway* (FDR=6.36E-03; with leading risk genes of *ADORA2B*, *CRHR1*, *GAK*, and *SH3GL2*) is relevant to striatal cAMP regulation and motor loop imbalance in PD^75^. The pathway of *Huntington disease risk genes* (FDR=1.42E-02; with leading risk genes of *GAK*, *HEXIM1*, *MAPT*, *NCOR1*, and *UBE2I*), converges with PD on basal ganglia circuitry, reflecting shared vulnerability of striatal pathways^76^. The pathway of *T cell activation* (FDR=2.18E-02; with leading risk genes of *CD38*, *MAPT*, *NSF*, *SH3GL2*, and *UBE2I*) indicates immune dysregulation that promotes neuroinflammation and dopaminergic loss^77^. The *TGF-beta signaling pathway*^78^ (FDR=4.33E-02; with leading risk genes of *MAPT*, *NCOR1*, *UBE2I*), is important for dopaminergic neuron survival and glial immune balance in PD-related neuroinflammation^78^. The *p38 MAPK* pathway (FDR=4.61E-02; with leading risk genes of *MAPT* and *UBE2I*) links microglial activation and oxidative stress to PD-related neurodegeneration^79^.

A total of 11 of 17 (64.7%) PWAS-O risk genes were interconnected in the PPI network plot (Fig. 4B). Notable genes include *GPNMB* and *SNX17*^53^ that have been linked to neuroinflammation and PD risk –– *GPNMB* has been presented as a PD risk gene that is elevated in PD plasma associating with PD severity^80^. The pathway enrichment analysis with PWAS-O risk genes (Fig. 4C) highlighted the *CCKR signaling map* (FDR=1.41E-02; with leading risk genes of *CD38*, *GFAP*, *RPS2*, and *SNX17*) that regulates dopamine release and glial inflammation^81^; the *ATP synthesis* pathway (FDR=1.42E-02; with leading risk genes of *HEXIM1* and *PDHB*) that reflects mitochondrial dysfunction in PD^82^; and the *T cell activation*^77^ pathway (FDR=1.93E-02; with leading risk genes of *CD38*, *PDHB*, and *SNX17*).

A total of 39 of 66 (59.1%) cell-type-aware TWAS-O risk genes in all six brain cell types formed a big interconnected PPI network, featuring well-established PD risk genes such as *MAPT*^27^, *SNCA*, and *LRRC37A2*^29^ as hubs (Fig. 5A). The corresponding pathway enrichment analysis (Fig. 5B) revealed significant pathways of *Huntington disease*^76^ (FDR=3.11E-03; with leading risk genes of *ARHGAP27*, *EFTUD2*, *HEXIM1*, *MAP3K14*, *MAPT*, *NCOR1*, *NMT1*, and *SNCA*); *T cell activation*^77^ (FDR=2.16E-02; with leading risk genes of *ARHGAP27*, *CD38*, *MAP3K14*, *MAPT*, *NSF*, and *SNCA*); *Synaptic vesicle trafficking* (FDR=2.90E-02; with leading risk genes of *NSF*, *SNCA*) and *Adrenaline and noradrenaline biosynthesis* pathway (FDR=2.95E-02; with leading risk genes of *NSF*, *SNCA*) that are both closely linked to PD-related neurotransmitter dysfunction^83,84^; CCKR signaling map^81^ (FDR=3.06E-02; with leading risk genes of *ARHGAP27*, *CD38*, *CDC27*, *GFAP*, *MAP3K14*, *MAPT*, *NCOR1*, *SNCA*); and *Parkinson disease* (FDR=3.29E-02; with leading risk genes of *EFTUD2*, *ERCC8*, *FAM13A*, *HEXIM1*, *MAP3K14*, *MAPT*, and *SNCA*).

Especially, T-cell activation pathway is enriched with risk genes detected by omnibus xWAS using bulk RNA-seq, snRNA-seq, and proteomics reference data; the CCKR signaling map pathway is enriched in the xWAS-O risk genes based on both proteomics and snRNA-seq reference data; and the Huntington disease pathway is enriched in the xWAS-O risk genes based on both bulk RNA-seq and snRNA-seq reference data. These overlapped pathways suggest shared immune and neurotransmission processes underlying PD.

## Discussion

### Cell-type-aware TWAS-O risk genes validated in bulk transcriptomics and proteomics

We integrated large-cohort of bulk RNA-seq^85^ (n=931), snRNA-seq^31^ (n=415), and bulk TMT proteomics data^86^ (n=716) of DLPFC with the latest GWAS summary data of PD^2^ through the xWAS-O framework^22^, with the goal of mapping risk genes of PD whose genetic effects are potentially mediated through bulk or cell-type-aware gene expressions or protein abundances in DLPFC. As a result, we identified 39 significant risk genes by bulk TWAS-O, 66 by cell-type-aware TWAS-O, and 21 by PWAS-O, which were respectively fine-mapped to 19, 32, and 11 independent signals (**Fig S14**). Comparing TWAS-O findings to the PWAS-O findings, we validated 11 out of 19 (57.9%) independent bulk TWAS-O signals, and 20 out of 32 (62.5%) independent cell-type-aware TWAS-O signals, that have overlapped test eQTLs with the test pQTLs of at least one of the significant PWAS-O risk genes. These results show that majority of TWAS-O risk genes have genetic effects mediated through both genetically regulated gene expressions and protein abundances.

Our cell-type-aware TWAS-O analysis yielded independent association signals largely concordant with bulk TWAS-O findings, demonstrating that majority PD associated risk genes might have genetic effects mediated through gene expressions in >1 brain cell types in DLPFC (see Table 4). Pairwise comparisons across six cell types also revealed substantial overlaps in the independent cell-type-aware TWAS-O findings (**Fig. S13**). A previous stratified-LDSC analysis integrating snRNA-seq study across multiple brain cell types including astrocytes, microglia, and oligodendrocytes etcs^87^ also showed that Parkinson’s disease risk loci are not enriched in genes specific to individual brain cell types or regions, but rather in gene sets involved in global cellular processes, especially those related to lysosomal function and genes intolerant to loss-of-function mutations, which are broadly expressed across multiple glial and neuronal subtypes. This previous study also showed that PD heritability did not localize to a single brain cell type but instead enriched in global processes of lysosomal, autophagy and LoF-intolerant pathways^87^.

### Cell-type-specific and novel TWAS-O signals were mapped

Nevertheless, we still identified a subset of independent signals that are cell-type-specific, e.g., *ACBD3*, *CLEC3B*, and *EIF2S2* in astrocytes, *TOX3* and *NCOR1* in Opcs. Especially, *ACBD3* and *CLEC3B* are also novel findings in astrocytes, linking PD genetic risks in pathways involving Golgi-mediated lipid trafficking and lectin-dependent neuroprotective regulation that could modulate dopaminergic resilience. In Opcs, *TOX3* and *NCOR1* are also novel findings that implicate chromatin remodeling and transcriptional repression in myelin-lineage vulnerability, an aspect of PD biology that has received comparatively little attention.

### TWAS-O/PWAS-O risk genes replicated in ROS/MAP

TWAS-O/PWAS-O risk genes detected using PD GWAS summary data^2^ were subjected to follow-up replication TWAS-O/PWAS-O analyses using individual level global parkinsonian score and pathologic PD traits and WGS genotype data in ROS/MAP cohort. The individual TWAS-O/PWAS-O results in ROS/MAP further supported the relevance of several prioritized risk genes to parkinsonism (8 unique genes) and neuropathologic manifestations of PD (24 unique genes), including *OTOF*, *CLEC3B*, *ACOX2*, *BST1*, *KHK*, *ACBD3*, and *VPS26B* that were highlighted in our primary TWAS-O/PWAS-O results with PD relevant biological functions. As expected, more genes are related to pathologic PD than the parkinsonism that quantifies motor functions. The TWAS-O/PWAS-O results in ROS/MAP provide additional evidence linking the TWAS-O/PWAS-O risk genes detected from publicly available large-scale PD GWAS summary data with refined motor function trait and PD pathology.

### PPI and pathway analyses illustrate biological functions of TWAS-O/PWAS-O signals

Further PPI network analyses of our bulk TWAS-O, cell-type-aware TWAS-O, and PWAS-O risk genes detected inter-connected PPI networks with hub genes of known PD risk genes, such as *MAPT*^27^, *SNCA*^28^, and *LRRC37A*^29^. Interestingly, gene pathway enrichment analyses identified several PD-related pathways that were significantly enriched with xWAS-O findings based on multiple reference omics data –– *T-cell activation*, *CCKR signaling map*, and *Huntington Disease*. Collectively, these findings emphasize both shared and complementary insights from different omics layers in shaping PD risk, through common gene pathways.

### Limitations of this study

Despite the strength of this study, it has several important limitations common to all TWAS and PWAS studies that rely on GWAS summary statistics. We observed genomic inflation in the findings using all three omics panels. To account for the inflation issue, we implemented the GIFT tool to identify independent signals, and used the bulk PWAS-O findings to validate significant TWAS-O risk loci. Second, the reference transcriptomics and proteomics data were profiled from a single prefrontal cortical region and it would be important to profile more than one site including other sites within and outside the brain given the widespread sites that accumulate PD pathology. Our study did not account for risk genes that act primarily in other PD-relevant brain tissues such as substantia nigra dopamine neurons or peripheral tissues. Consequently, it is unclear to what extent the phenotypic heterogeneity of PD depends on differences in sites specific underlying genetics differences. However, our study demonstrated the feasibility of integrating multi-omics data with GWAS summary data to map risk genes for PD. The xWAS-O procedure can be easily applied to integrate omics data profiled from other brain regions and GWAS data to map risk genes of PD and other complex diseases. Finally, functional follow-up in cellular and in-vivo systems will be essential to reveal the underlying biological mechanisms of detected risk genes.

### Conclusions

In summary, we demonstrated that by integrating large-scale bulk RNA-seq, snRNA-seq, and TMP proteomics data of DLPFC with the latest GWAS summary data^2^ of PD through the xWAS-O framework yielded a thorough view of the risk genes of PD. We identified TWAS risk genes that were linked to PD-related traits in GWAS Catalogue, as well as novel risk genes, especially novel cell-type-specific risk genes, that we were able to validate in the proteomics data from the same DLPFC tissue and replicated in individual-level GWAS data of ROS/MAP. From a translational perspective, our results provide a prioritized list of bulk and cell-type-aware candidate TWAS risk genes for functional validation and therapeutic targeting. Our findings highlight the importance of integrative multi-omics data and GWAS summary data in unraveling the complex molecular mechanisms underlying human diseases like PD.

## Methods

### xWAS-O analytical framework

The xWAS-O pipeline recently developed by our group^19^ can integrate multi-omics data (i.e., bulk-RNAseq, sn-RNAseq, and bulk proteomics) with GWAS summary data using multiple complementary statistical methods. In brief, Stage I of xWAS-O trains cis-xQTL imputation models using individual-level genotype and transcriptomic/proteomic expression data. Stage II performs gene-based association testing by integrating estimated xQTL weights with GWAS summary statistics data, followed by omnibus p-values aggregation through ACAT^88^. This framework has been shown to improve statistical power and maintain well-calibrated type I error rates^17,23^.

#### Stage I. xQTL weight estimation.

As described in our previous studies^17^, for each gene and for each corresponding molecular trait, cis-xQTL weights were estimated using genotype data (G) within ±1 Mb of the transcription start/termination site as predictors. The target molecular trait was first adjusted for known covariates (e.g., age, sex, batch effects, PEER factors^89^ and genotype PCs) and log2-transformed, where the residuals were used as the quantitative traits ($T_{g}$). Three complementary statistical methods were implemented to estimate cis-xQTL weights (Equation 1): (i) Nonparametric DPR implemented in TIGAR^6,7^, (ii) Penalized regression with EN penalty implemented in PrediXcan^4^; (iii) the best predictive model selected from FUSION, which considers penalized regression with LASSO, EN, BLUP, and Top1-QTL^5^.


(Equation 1) $$T_{g}=\text{Gw}+\epsilon ;\;\epsilon \sim N\left(0,\sigma_{\epsilon }^{2}I\right).$$

#### Stage II. Gene-based Association testing.

Genes with CV $R^{2}>0.5\%$ for their corresponding molecular traits were retained for association testing. As shown by the TIGAR-V2 paper^7^, a relaxed threshold does not inflate type I error, because TWAS Z-scores are linear combinations of GWAS statistics weighted by estimated eQTL effects, imprecise weights reduce power but do not introduce bias.

In Stage II, xWAS-O first uses these three sets of xQTL weights ($\hat{w}$) in Equation 1 from Stage I to predict the genetically regulated genetic component of expression (GReX) in the GWAS test data by $G_{\text{gwas}}w$, and then test the association between the phenotype Y and the GReX component as follows:

(Equation 2) $$f(E[Y])=\alpha \left(G_{\text{gwas}}\hat{w}\right);\;H_{o}:\alpha =0,\;H_{a}:\alpha \neq 0.$$

When individual genotype data of the GWAS cohort ($G_{\text{gwas}}$) are not available, it is equivalent to take $\hat{w}$ as variant weights to implement gene-based burden test as shown in the S-PrediXcan paper^90^. A gene-based association Z-score test statistic can be calculated using the summary-level GWAS test data as follows:

(Equation 3) $${\tilde{Z}}_{S_{-}\text{PrediXcan}}=\frac{\sum_{\text{l}=1}^{\text{m}}\left(\hat{w_{l}}\hat{\sigma_{l}}Z_{l}\right)}{\sqrt{{\hat{w}}^{\prime }V\hat{w}}};\;\hat{\sigma_{l}^{2}}=\text{Var}\left(G_{0,1}\right);\;V=\text{Cov}\left(G_{0}\right).$$

Here, $Z_{l}$ denotes the single-variant Z-score test statistics in GWAS summary data for the $l^{\text{th}}$ genetic variant, $\hat{\sigma_{l}^{2}}$ denotes genotype variance of the $l^{\text{th}}$ genetic variant, and V denotes the genotype variance-covariance matrix. Both $\hat{\sigma_{l}^{2}}$ and V could be approximated from individual-level genotype data of an external reference panel of the same ancestry as the test GWAS data (with genotype matrix $G_{0}$).

Reference LD derived from the WGS genotype data of the ROS/MAP cohorts^30^ was used for deriving xWAS Z-score test statistics in this study. Gene-based association test (two-sided) p-values can be easily derived from the Z-score statistic in Equation 3 that has a standard normal distribution under the null hypothesis of no association exists between genetically regulated molecular traits and the phenotype of interest.

#### Derive omnibus xWAS p-values.

As shown by our previous studies^23,91^, there is no single statistical method that can achieve optimal performance of modeling the underlying genetic architecture of genome-wide genes. Thus, to enhance the robustness and statistical power of gene-based association testing, xWAS-O utilizes the ACAT^34^ to combine p-values from all three imputation tools based on complementary statistical methods as:

(Equation 4) $$T_{\text{ACAT}}=\frac{1}{k}\sum_{k=1}^{K=3}\text{tan}\left[\frac{\pi }{2}\left(0.5-p_{k}\right)\right];p\in \left[0,1\right].$$

Here, K denotes the total number of xWAS p-values per gene, and $p_{k}$ denotes the p-value obtained from a gene-based association test by each of these three tools. The ACAT test statistic ($T_{\text{ACAT}}$) approximately follows a standard Cauchy distribution^19^ under the null hypothesis.

An omnibus test p-value (two-sided) for each test molecular trait of a test gene can be calculated by Equation 5 and is then used to identify significant xWAS risk genes^23^.

(Equation 5) $${\text{pvalue}}_{T}\approx 0.5-\frac{\left\{\text{arctan}\left(T\right)\right\}}{\pi },\;\pi \approx 3.14.$$

By comprehensive simulation studies and real studies of complex traits, previous studies^23,91^ have shown that improved power and calibrated type I error rates were obtained using ACAT omnibus xWAS p-values.

### Fine-map xWAS-O risk genes by GIFT

To fine-map risk genes within genomic regions containing multiple significant xWAS-O signals, we applied Gene-based Integrative Fine-mapping through conditional TWAS, referred to as GIFT^24^. First, for each candidate locus containing multiple significant xWAS-O risk genes, we considered the merged test regions of all nearby significant xWAS-O risk genes whose test regions were overlapped with the top significant risk gene. GIFT then jointly models the genetically regulated expression or protein abundance of all significant xWAS-O risk genes in the region, explicitly accounting for both LD structure and correlations among predicted molecular traits. As a result, GIFT can effectively pinpoint conditionally independent signals.

Here, we briefly describe the underlying model assumptions by GIFT for using summary xQTL and GWAS data. For each gene i, the marginal association Z-scores from the xQTL data can be modeled as:

(Equation 6) $${\hat{Z}}_{T_{i}}=\sqrt{\left(n_{1}-1\right)}{𝚺}_{1i}w_{i}+\epsilon_{T_{i}},i=1,\ldots ,k.$$

Here, $n_{1}$ is the sample size for xQTL summary data, ${𝚺}_{1i}$ is the LD correlation matrix of cis-SNPs for gene $i,w_{i}$ represents the vector of cis-SNP effect sizes on gene expression or protein abundance as in Equation 1, and $\epsilon_{T_{i}}$ captures residual errors.

The marginal Z-score statistics of all cis-SNPs in the target genetic region from the GWAS summary data can be modeled as:

(Equation 7) $${\overset{ˆ}{Z}}_{y}=\sqrt{\left(n_{2}-1\right)}{𝚺}_{2}{\left(\alpha_{1}w_{1}^{T},\ldots ,\alpha_{k}w_{k}^{T}\right)}^{T}+\epsilon_{y}.$$

Here, $n_{2}$ is the sample size for GWAS summary data; ${𝚺}_{2}$ is the LD correlation matrix of all cis-SNPs; $w_{i}$ are xQTL effect sizes defined in Equation 6; $\alpha_{i}$ denotes the conditional effect of the GReX or genetically regulated protein abundance (GRP) of gene i on the phenotype, while accounting for the genetic components of all neighboring genes $\left(\alpha_{-i}\right)$ in the same multiple linear regression model; and $\epsilon_{y}$ represents residual errors. A gene with significant conditional effect $\alpha_{i}$ will be the independent signal prioritized by GIFT. A gene with most significant marginal TWAS-O p-value may not always be the one prioritized by GIFT in a joint model of all significant genes in the same target genetic region as in Equation 7.

Since our omnibus framework does not provide a combined xQTL weights, we implemented GIFT with xQTL and GWAS summary data. We generated xQTL summary data (${\hat{Z}}_{T_{i}}$) and the corresponding LD (${𝚺}_{1i}$) from the individual-level snRNA-seq, TMT proteomics, and whole genome sequencing data of ROS/MAP samples^30^. We used GWAS LD $\left({𝚺}_{2}\right)$ derived from the UK Biobank^92^ reference panel. Reference LD derived from different reference panels are recommended by GIFT developers for resolving potential computational issues.

### PPI network analyses

Protein-protein interaction (PPI) networks were constructed for significant risk genes using the STRING^25,73,93^ webtool (version 12.0), which integrates public data sources of protein-protein interaction information to construct connected networks of proteins (nodes in the PPI network). Protein-protein edges represent the predicted functional associations, colored differently to indicate seven categories –– co-expression, text-mining, experiments (biochemical/genetic data), databases (previously curated pathway and protein complex information), gene co-occurrence, gene fusion, and neighborhood. Gene co-occurrence, fusion, and neighborhood represent predicted association based on genome-wide comparisons. Each edge in the network represents a predicted functional association. Lists of significant risk genes by bulk TWAS-O, cell-type-aware TWAS-O, and PWAS-O analyses were submitted to the STRING webtool to assess their corresponding PPI network connectivity.

### Enrichment analyses by pathDIP

PathDIP 5^94^ integrates pathways from 6,500-plus curated records across many databases, unifying them with a KEGG-/Reactome-based ontology so enrichment results can be filtered, consolidated, and mapped into 53 functional categories, with FDR q-values calculated by the Benjamini-Hochberg method. Specific pathway sources can be selected for gene enrichment analyses which aim to detect pathways that are significantly enriched with the curated gene list versus a similar list of random genes. We utilized the pathDIP webtool to conduct gene pathway enrichment analyses with respect to data bases of Panther^95^, PathBank^96^, and Reactome^97^ with the lists of bulk TWAS-O, cell-type-aware TWAS-O, and PWAS-O risk genes of PD.

### Global parkinsonian score and pathologic PD traits in ROS/MAP

As described in previous publication about studying parkinsonism and PD pathologies in ROS/MAP older adults^64^, the global parkinsonian score is profiled annually by trained nurse clinicians through assessing four parkinsonian signs (parkinsonian gait, rigidity, bradykinesia, and tremor) using 26 items from a modified Unified Parkinson’s Disease Rating Scale^71,72^; the pathologic PD is a binary variable defined as either nigral neuronal loss or Lewy body pathology exists.

A score for each of the four parkinsonian signs was based on the sum of the scores for each of its individual items assessed, e.g., 8 items for bradykinesia. The scores for the 4 parkinsonian signs were averaged to provide a continuous global parkinsonian score as previously described.^71^ The distribution of global parkinsonian score was positively skewed and was square root transformed prior to our analyses.

Nigral neuronal loss was assessed in the substantia nigra in a hemisection of the mid to rostral midbrain near or at the exit of the 3rd nerve in hematoxylin and eosin stained 6um sections using a semi-quantitative scale (0–3). Lewy body was defined as either existing in one of the six regions (substantia nigra, anterior cingulate cortex, entorhinal cortex, midfrontal cortex, superior or middle temporal cortex, inferior parietal cortex), which were assessed using a monoclonal phosphorylated antibody to a-synuclein (1:20,000; Wako Chemical USA Inc., Richmond, VA).

## Supplementary Material

Supplementary Files

This is a list of supplementary files associated with this preprint. Click to download.

- SupplementaryInformationMay2026.docx
- SupplementaryDataMay2026.xlsx

Supplementary data 1–10 in the excel data file and Fig. S1–14 are included.

## Figures and Tables

**Table 1:** Significant risk genes by bulk TWAS-O in DLPFC. Superscript a denotes a gene is reported as associated with PD in GWAS Catalog. A risk gene is considered as replicated by PWAS-O if it is either detected as significant by PWAS-O or has test xQTLs overlapped with the ones of a significant bulk PWAS-O risk gene, denoted by superscript b. Independent significant genes noted here are either not overlapping with other bulk TWAS-O risk genes or fine-mapped by GIFT, denoted by c. Independent risk genes that are not subjected to GIFT fine-mapping have NA GIFT p-values. Gene names of bulk TWAS-O risk genes that are independent signals and not reported as associated with PD in GWAS Catalog are bold.

Gene Name	CHR	Start Position	TWAS p-values	GIFT p-values	Gene Name	CHR	Start Position	TWAS p-values	GIFT p-values
EFNA3^a,c^	1	155078872	6.00e-07	NA	ACBD4^b^	17	45132600	1.01e-18	1.00
RAB29^a,c^	1	205767986	8.53e-09	NA	**ADORA2B** ^b,c^	17	15944917	5.41e-07	2.28e-02
**ACOX2** ^b,c^	3	58505136	8.99e-08	NA	ARL17A^b^	17	46516702	6.33e-07	6.24e-01
CD38^a,b,c^	4	15778275	3.82e-16	NA	CRHR1^a,b^	17	45784280	5.38e-20	1.00
GAK^a,c^	4	849276	3.37e-10	4.31e-05	HEXIM1^b^	17	45148502	1.18e-19	1.00
IDUA	4	986997	4.95e-10	1.61e-01	LINC02210^a,b,c^	17	45620328	6.94e-20	1.31e-05
MMRN1^a,c^	4	89879532	8.38e-18	NA	LRRC37A^b^	17	46292733	8.66e-08	5.11e-02
SCARB2^a,c^	4	76158733	1.63e-10	NA	LRRC37A17P^b^	17	46978481	2.11e-18	1.00
TMEM175^a^	4	932387	5.36e-07	1.13e-01	LRRC37A2^b^	17	46511511	7.20e-10	1.00
NDUFAF2^a,c^	5	60945129	1.01e-07	NA	LRRC37A4P^b^	17	45506741	2.57e-20	1.00
GPNMB^a,b,c^	7	23235967	8.83e-09	5.46e-03	MAPT^a,b^	17	45894382	4.94e-20	1.00
**NUPL2** ^b,c^	7	23181827	7.70e-08	8.53e-03	MAPT-AS1^a,b^	17	45799390	1.44e-07	1.00
CTSB^a,b,c^	8	11842524	1.65e-06	NA	MAPT-IT1^b^	17	45895783	1.86e-20	1.00
SH3GL2^a,c^	9	17579082	1.24e-06	NA	**NCOR1** ^b,c^	17	16029157	6.70e-07	4.36e-02
**ABCB9** ^c^	12	122920951	7.16e-07	NA	NSF^a,b,c^	17	46590669	9.04e-20	3.11e-02
**MRPS34** ^b,c^	16	1771890	7.29e-08	1.26e-04	PLCD3^b^	17	45108967	9.28e-10	1.00
TSR3^b^	16	1349240	1.79e-06	4.42e-01	TTC19^b^	17	15999380	1.13e-06	7.97e-01
UBE2I^b^	16	1308880	1.42e-06	4.47e-01	WNT3^a,b^	17	46762506	1.29e-19	1.00
**AC005670.2** ^b,c^	17	46923133	1.35e-17	1.37e-02	ZSWIM7^b^	17	15976560	3.76e-07	2.42e-01
AC142472.1^b^	17	45146730	2.30e-12	1.00					

a. Known risk genes reported in GWAS Catalogue.

b. Replicated by PWAS-O.

c. Independent bulk TWAS-O risk genes.

**Table 2:** Independent significant cell-type-aware TWAS-O risk genes in DLPFC. All significant genes listed in this table are independent risk genes that either not overlapping with other nearby significant cell-type-aware TWAS-O risk genes, or fine-mapped by GIFT. Independent risk genes that are not subjected to GIFT fine-mapping have NA GIFT p-values. Superscript a denotes a gene is reported as associated with PD in GWAS Catalog. A risk gene is considered as overlapped with significant bulk TWAS-O (or PWAS-O) risk genes if it is either detected as significant by bulk TWAS-O (or PWAS-O) or has test xQTLs overlapped with the ones of a significant bulk TWAS-O (or PWAS-O) risk gene, denoted by superscript b (or c). A risk gene is considered as cell-type-specific if it is only detected as significant by this cell type, denoted by superscript d. Independent risk genes that are not reported as associated with PD in GWAS catalog or bulk TWAS-O are bold.

Gene Name	CHR	Cell type	TWAS p-values	GIFT p-values	Gene Name	CHR	Cell type	TWAS p-values	GIFT p-values
**ACBD3** ^d^	1	Ast	3.81e-07	NA	GPNMB^a,b,c^	7	Ex, In, Mic	7.60e-08, 2.33e-07, 2.41e-08	NA, NA, NA
**KHK** ^c^	2	Mic	2.26e-06	NA	C16orf91^b,c^	16	Oli	6.45e-07	NA
**OTOF** ^c^	2	In	7.15e-07	1.61e-03	FAHD1^b,c^	16	Ast, Opc	6.28e-08, 1.26e-07	NA, NA
TMEM163^a^	2	In, Mic	6.64e-07, 2.16e-11	NA, NA	NME3^b,c^	16	In	1.63e-07	5.23e-05
**CLEC3B** ^c,d^	3	Ast	1.69e-06	NA	SPSB3^b,c^	16	Ex, Mic	1.55e-07, 1.55e-07	5.02e-14, 1.82e-02
**GPR171**	3	Ex, Opc	1.88e-06, 2.60e-07	NA, NA	**TOX3** ^d^	16	Opc	1.36e-06	NA
BST1^a,b,c^	4	Mic	1.06e-06	NA	C1QL1^b,c^	17	Ast	6.32e-17	1.29e-02
CC2D2A^a,b,c^	4	In	4.75e-07	NA	DCAKD^b,c^	17	In	2.84e-19	1.50e-24
CCSER1^a,b^	4	In, Opc	6.26e-08, 6.55e-07	1.06e-02, 1.67e-08	GFAP^b,c^	17	Ast	7.24e-08	6.97e-03
CD38^a,b,c^	4	Ex, Oli	4.41e-10, 4.73e-13	NA, NA	LRRC37A^b,c^	17	Ast	6.23e-12	3.64e-02
FAM13A^b^	4	Mic, Opc	5.01e-09, 1.17e-09	3.59e-36, 3.45e-06	LRRC37A2^b,c^	17	Ast	1.30e-10	1.72e-02
FGFRL1^b^	4	Ex	3.01e-15	NA	NCOR1^b,c,d^	17	Opc	2.36e-06	NA
GPRIN3^a,b^	4	Ast	1.75e-07, 9.75e-07	NA, NA	PLEKHM1^a,b,c^	17	In	1.49e-09	2.23e-16
NELFA^b^	4	Opc	2.36e-06	NA	RPRML^b,c^	17	Mic, Opc	2.21e-16, 3.22e-20	5.03e-10, 6.35e-03
ERCC8^b^	5	Ast	1.43e-09	NA	WNT3^a,b,c^	17	Oli	5.32e-08	4.25e-02
ZSWIM6^b^	5	Mic	1.30e-08	NA	**EIF2S2** ^d^	20	Ast	1.00e-06	NA

a. Known risk genes reported in GWAS Catalogue.

b. Overlapped with significant bulk TWAS-O risk genes.

c. Replicated by bulk PWAS-O.

d. Cell-type-specific.

**Table 3:** Significant PWAS-O risk genes in DLPFC. Superscript a denotes a gene is reported as associated with PD in GWAS Catalog. PWAS-O risk genes that are also overlapped with bulk TWAS-O risk genes are denoted by superscript b. Independent PWAS-O risk genes that are either not overlapping with other nearby significant PWAS-O risk genes, or fine-mapped by GIFT, are denoted by superscript c. All PWAS q-values were obtained from p-values adjusted by genomic control factor. Independent risk genes that are not subjected to GIFT fine-mapping have NA GIFT p-values. Gene names of bulk PWAS-O risk genes that are fine-mapped independent signals, not reported as associated with PD in GWAS Catalog or detected by our bulk TWAS-O analyses, are bold.

Gene Name	CHR	Start Position	PWAS p-values	PWAS q-values	GIFT p-values
**SNX17** ^c^	2	27370496	1.28e-05	6.73e-03	NA
**EXOSC7** ^c^	3	44976249	2.35e-06	1.58e-03	NA
FAM107A^b,c^	3	58564112	1.27e-04	3.76e-02	8.64e-16
PDHB^b^	3	58427630	8.72e-05	3.75e-02	9.37e-01
CD38^a,b,c^	4	15778308	3.30e-14	5.19e-11	NA
GPNMB^a,b,c^	7	23246697	4.20e-08	4.96e-05	NA
CTSB^a,b,c^	8	11842525	1.04e-04	3.76e-02	NA
SEC23IP^a,c^	10	119892573	1.62e-04	4.49e-02	NA
**VPS26B** ^c^	11	134224605	1.36e-06	1.07e-03	NA
MLST8^b^	16	2205177	1.26e-04	3.76e-02	8.01e-01
RAB26^b^	16	2148144	1.12e-04	3.76e-02	7.69e-01
RPS2^b^	16	1962061	7.68e-05	3.63e-02	1.73e-01
GFAP^b^	17	44905626	5.67e-06	3.35e-03	1.99e-01
GOSR2^b,c^	17	46923120	2.73e-14	5.19e-11	3.34e-08
HEXIM1^b^	17	45147317	8.58e-08	8.10e-05	2.77e-01
NMT1^b,c^	17	45061299	3.71e-17	1.75e-13	2.83e-10
TTC19^b,c^	17	15999380	1.15e-04	3.76e-02	NA

a. Known risk genes reported in GWAS Catalogue.

b. Overlapped with significant bulk TWAS-O risk genes.

c. Independent PWAS-O risk genes.

**Table 4:** Summary of cell-type-aware TWAS-O risk genes.

Cell Types	Number of significant cell-type-aware TWAS-O risk genes	Number of risk genes reported as associated with PD-related traits in GWAS Catalog	Number of independent cell-type-aware TWAS-O risk genes	Number of independent cell-typeaware TWAS-O risk genes overlapped with bulk TWAS-O	Number of independent cell-typeaware TWAS-O risk genes overlapped with PWAS-O	Number of independent cell-type-aware TWAS-O risk genes that are cell-type-specific
Astrocytes	21	5	10	7	6	3
Microglia	24	8	8	6	5	0
Excitatory neurons	25	6	5	4	3	0
Inhibitory neurons	19	9	8	6	6	0
Oligodendrocytes	21	9	4	4	3	0
Opcs	23	6	8	6	3	2

## Data Availability

All omics data of ROS/MAP samples are available from Synapse (https://www.synapse.org/Synapse:syn3219045), which can be requested through www.radc.rush.edu and www.synapse.org. GWAS summary data of PD by the Nalls et. al. 2019 exclude the 23andMe cohort are available from google drive (https://drive.google.com/drive/folders/10bGj6HfAXgl-JslpI9ZJIL_JIgZyktxn). Trained bulk cis-eQTL weights, cell-type-aware cis-eQTL weights, cis-pQTL weights are available from SYNAPSE (syn66644078; https://doi.org/10.7303/syn66644078). Omnibus TWAS/PWAS summary data of PD will be shared through SYNAPSE once this work is published.

## Code availability

Analysis scripts used to produce all results in this work is available at https://github.com/Leo-LiuQiang/CTS-TWAS.

## References

[1] Kalia L. V. & Lang A. E. Parkinson’s disease. The Lancet 386, 896–912 (2015). 10.1016/S0140-6736(14)61393-3 25904081

[2] Nalls M. A. Identification of novel risk loci, causal insights, and heritable risk for Parkinson’s disease: a meta-analysis of genome-wide association studies. The Lancet Neurology 18, 1091–1102 (2019). 10.1016/S1474-4422(19)30320-5 31701892 PMC8422160

[3] Kim J. J. Multi-ancestry genome-wide association meta-analysis of Parkinson’s disease. Nature Genetics 56, 27–36 (2024). 10.1038/s41588-023-01584-8 38155330 PMC10786718

[4] Gamazon E. R. A gene-based association method for mapping traits using reference transcriptome data. Nature Genetics 47, 1091–1098 (2015). 10.1038/ng.3367 26258848 PMC4552594

[5] Gusev A. Integrative approaches for large-scale transcriptome-wide association studies. Nature Genetics 48, 245–252 (2016). 10.1038/ng.3506 26854917 PMC4767558

[6] Nagpal S. TIGAR: An Improved Bayesian Tool for Transcriptomic Data Imputation Enhances Gene Mapping of Complex Traits. The American Journal of Human Genetics 105, 258–266 (2019). 10.1016/j.ajhg.2019.05.018 31230719 PMC6698804

[7] Parrish R. L., Gibson G. C., Epstein M. P. & Yang J. TIGAR-V2: Efficient TWAS tool with nonparametric Bayesian eQTL weights of 49 tissue types from GTEx V8. Human Genetics and Genomics Advances 3, 100068 (2022). 10.1016/j.xhgg.2021.100068 35047855 PMC8756507

[8] Yuan Z. Testing and controlling for horizontal pleiotropy with probabilistic Mendelian randomization in transcriptome-wide association studies. Nature Communications 11, 3861 (2020). 10.1038/s41467-020-17668-6 PMC7395774 32737316

[9] Yao S. A transcriptome-wide association study identifies susceptibility genes for Parkinson’s disease. npj Parkinson’s Disease 7, 79 (2021). 10.1038/s41531-021-00221-7 PMC8429416 34504106

[10] Shi J.-J. Joint analysis of proteome, transcriptome, and multi-trait analysis to identify novel Parkinson’s disease risk genes. Aging 16, 1555–1580 (2024). 10.18632/aging.205444 38240717 PMC10866412

[11] Kia D. A. Identification of Candidate Parkinson Disease Genes by Integrating Genome-Wide Association Study, Expression, and Epigenetic Data Sets. JAMA Neurology 78, 464–472 (2021). 10.1001/jamaneurol.2020.5257 33523105 PMC7851759

[12] Li Y. I., Wong G., Humphrey J. & Raj T. Prioritizing Parkinson’s disease genes using population-scale transcriptomic data. Nature Communications 10, 994 (2019). 10.1038/s41467-019-08912-9 PMC6397174 30824768

[13] Martirosyan A. Unravelling cell type-specific responses to Parkinson’s Disease at single cell resolution. Molecular Neurodegeneration 19, 7 (2024). 10.1186/s13024-023-00699-0 38245794 PMC10799528

[14] Bryois J. Genetic identification of cell types underlying brain complex traits yields insights into the etiology of Parkinson’s disease. Nature Genetics 52, 482–493 (2020). 10.1038/s41588-020-0610-9 32341526 PMC7930801

[15] Lee A. J. Characterization of altered molecular mechanisms in Parkinson’s disease through cell type–resolved multiomics analyses. Science Advances 9, eabo2467 10.1126/sciadv.abo2467 PMC10104466 37058563

[16] Andersen M. S. Heritability Enrichment Implicates Microglia in Parkinson’s Disease Pathogenesis. Annals of Neurology 89, 942–951 (2021). 10.1002/ana.26032 33502028 PMC9017316

[17] Hu T. Proteome-wide association studies using summary pQTL data of brain, CSF, and plasma identify 30 risk genes of Alzheimer’s disease dementia. Alzheimer’s Research & Therapy 17, 135 (2025). 10.1186/s13195-025-01774-y PMC12175411 40533815

[18] Liu Q. Cell-type-aware transcriptome-wide association studies identify 91 independent risk genes for Alzheimer’s disease dementia. Commun Biol (2026). 10.1038/s42003-026-10030-4 PMC13287724 42020711

[19] Nagpal S. TIGAR: An Improved Bayesian Tool for Transcriptomic Data Imputation Enhances Gene Mapping of Complex Traits. American journal of human genetics 105, 258–266 (2019). 10.1016/j.ajhg.2019.05.018 31230719 PMC6698804

[20] Gamazon E. R. A gene-based association method for mapping traits using reference transcriptome data. Nature genetics 47, 1091–1098 (2015). 10.1038/ng.3367 26258848 PMC4552594

[21] Gusev A. Integrative approaches for large-scale transcriptome-wide association studies. Nature genetics 48, 245–252 (2016). 10.1038/ng.3506 26854917 PMC4767558

[22] Hu T. Omnibus proteome-wide association study identifies 43 risk genes for Alzheimer disease dementia. American journal of human genetics 111, 1848–1863 (2024). 10.1016/j.ajhg.2024.07.001 39079537 PMC11393696

[23] Hu T. Omnibus proteome-wide association study identifies 43 risk genes for Alzheimer disease dementia. The American Journal of Human Genetics 111, 1848–1863 (2024). 10.1016/j.ajhg.2024.07.001 39079537 PMC11393696

[24] Liu L. Conditional transcriptome-wide association study for fine-mapping candidate causal genes. Nature Genetics 56, 348–356 (2024). 10.1038/s41588-023-01645-y 38279040

[25] Szklarczyk D. The STRING database in 2023: protein–protein association networks and functional enrichment analyses for any sequenced genome of interest. Nucleic Acids Research 51, D638–D646 (2023). 10.1093/nar/gkac1000 36370105 PMC9825434

[26] Rahmati S., Abovsky M., Pastrello C. & Jurisica I. pathDIP: an annotated resource for known and predicted human gene-pathway associations and pathway enrichment analysis. Nucleic Acids Research 45, D419–D426 (2017). 10.1093/nar/gkw1082 27899558 PMC5210562

[27] Pascale E. Genetic Architecture of MAPT Gene Region in Parkinson Disease Subtypes. Frontiers in Cellular Neuroscience Volume 10 - 2016 (2016). 10.3389/fncel.2016.00096 PMC4826864 27147968

[28] Siddiqui I. J., Pervaiz N. & Abbasi A. A. The Parkinson Disease gene SNCA: Evolutionary and structural insights with pathological implication. Scientific Reports 6, 24475 (2016). 10.1038/srep24475 27080380 PMC4832246

[29] Bowles K. R. 17q21.31 sub-haplotypes underlying H1-associated risk for Parkinson’s disease are associated with LRRC37A/2 expression in astrocytes. Molecular Neurodegeneration 17, 48 (2022). 10.1186/s13024-022-00551-x 35841044 PMC9284779

[30] Bennett D. A. Religious Orders Study and Rush Memory and Aging Project. Journal of Alzheimer’s Disease 64, S161–S189 (2018). 10.3233/JAD-179939 PMC6380522 29865057

[31] Fujita M. Cell subtype-specific effects of genetic variation in the Alzheimer’s disease brain. Nature Genetics 56, 605–614 (2024). 10.1038/s41588-024-01685-y 38514782 PMC12288883

[32] Mathys H. Single-cell atlas reveals correlates of high cognitive function, dementia, and resilience to Alzheimer’s disease pathology. Cell 186, 4365–4385.e4327 (2023). 10.1016/j.cell.2023.08.039 37774677 PMC10601493

[33] Olah M. Single cell RNA sequencing of human microglia uncovers a subset associated with Alzheimer’s disease. Nature Communications 11, 6129 (2020). 10.1038/s41467-020-19737-2 PMC7704703 33257666

[34] Liu Y. ACAT: A Fast and Powerful p Value Combination Method for Rare-Variant Analysis in Sequencing Studies. American journal of human genetics 104, 410–421 (2019). 10.1016/j.ajhg.2019.01.002 30849328 PMC6407498

[35] Devlin B. & Roeder K. Genomic Control for Association Studies. Biometrics 55, 997–1004 (1999). 10.1111/j.0006-341X.1999.00997.x 11315092

[36] Benjamini Y. & Hochberg Y. Controlling the false discovery rate: a practical and powerful approach to multiple testing. Journal of the Royal statistical society: series B (Methodological) 57, 289–300 (1995).

[37] Buniello A. The NHGRI-EBI GWAS Catalog of published genome-wide association studies, targeted arrays and summary statistics 2019. Nucleic Acids Research 47, D1005–D1012 (2019). 10.1093/nar/gky1120 30445434 PMC6323933

[38] Sui J. S. Y. Altered expression of ACOX2 in non-small cell lung cancer. BMC Pulmonary Medicine 22, 321 (2022). 10.1186/s12890-022-02115-7 35999530 PMC9396774

[39] Wu Y.-S. Joint-tissue integrative analysis identifies high-risk genes for Parkinson’s disease. Frontiers in Neuroscience Volume 18 - 2024 (2024). 10.3389/fnins.2024.1309684 PMC10991821 38576865

[40] Lake N. J. Biallelic Mutations in MRPS34 Lead to Instability of the Small Mitoribosomal Subunit and Leigh Syndrome. The American Journal of Human Genetics 101, 239–254 (2017). 10.1016/j.ajhg.2017.07.005 28777931 PMC5544391

[41] Bustos B. I. Genome-wide contribution of common short-tandem repeats to Parkinson’s disease genetic risk. Brain 146, 65–74 (2023). 10.1093/brain/awac301 36347471 PMC10060720

[42] O Brien H. E. Expression quantitative trait loci in the developing human brain and their enrichment in neuropsychiatric disorders. Genome Biology 19, 194 (2018). 10.1186/s13059-018-1567-1 30419947 PMC6231252

[43] Wang J., Hoshino T., Redner R. L., Kajigaya S. & Liu J. M. ETO, fusion partner in t(8;21) acute myeloid leukemia, represses transcription by interaction with the human N-CoR/mSin3/HDAC1 complex. Proceedings of the National Academy of Sciences 95, 10860–10865 (1998). 10.1073/pnas.95.18.10860 PMC27986 9724795

[44] Fan J., Liu J., Culty M. & Papadopoulos V. Acyl-coenzyme A binding domain containing 3 (ACBD3; PAP7; GCP60): An emerging signaling molecule. Progress in Lipid Research 49, 218–234 (2010). 10.1016/j.plipres.2009.12.003 20043945 PMC2873055

[45] Chen Z. Tetranectin gene deletion induces Parkinson’s disease by enhancing neuronal apoptosis. Biochemical and Biophysical Research Communications 468, 400–407 (2015). 10.1016/j.bbrc.2015.10.118 26597345

[46] Mutez E. Involvement of the immune system, endocytosis and EIF2 signaling in both genetically determined and sporadic forms of Parkinson’s disease. Neurobiology of Disease 63, 165–170 (2014). 10.1016/j.nbd.2013.11.007 24269915

[47] Li Y. Ketohexokinase-dependent metabolism of cerebral endogenous fructose in microglia drives diabetes-associated cognitive dysfunction. Experimental & Molecular Medicine 55, 2417–2432 (2023). 10.1038/s12276-023-01112-y 37907746 PMC10689812

[48] Fujiwara Y. The GPR171 pathway suppresses T cell activation and limits antitumor immunity. Nature Communications 12, 5857 (2021). 10.1038/s41467-021-26135-9 PMC8494883 34615877

[49] Lindberg I. The proSAAS Chaperone Provides Neuroprotection and Attenuates Transsynaptic α-Synuclein Spread in Rodent Models of Parkinson’s Disease. Journal of Parkinson’s Disease 12, 1463–1478 (2022). 10.3233/JPD-213053 PMC9731515 35527562

[50] Tang L. A primate nigrostriatal atlas of neuronal vulnerability and resilience in a model of Parkinson’s disease. Nature Communications 14, 7497 (2023). 10.1038/s41467-023-43213-2 PMC10657376 37980356

[51] Margolis R. L. cDNAs with long CAG trinucleotide repeats from human brain. Human Genetics 100, 114–122 (1997). 10.1007/s004390050476 9225980

[52] Mohtashami S. TOX3 Variants Are Involved in Restless Legs Syndrome and Parkinson’s Disease with Opposite Effects. Journal of Molecular Neuroscience 64, 341–345 (2018). 10.1007/s12031-018-1031-4 29404899

[53] Zhang H. The Retromer Complex and Sorting Nexins in Neurodegenerative Diseases. Frontiers in Aging Neuroscience Volume 10 - 2018 (2018). 10.3389/fnagi.2018.00079 PMC5879135 29632483

[54] Seong J. B., Kim Y.-H., Huh J.-W. & Lee Y. VPS26B PLAYS A PROTECTIVE ROLE AGAINST MPP+-INDUCED NEUROTOXICITY IN DOPAMINERGIC SH-SY5Y CELLS. IBRO Neuroscience Reports 15, S203 (2023). 10.1016/j.ibneur.2023.08.317

[55] Jaccard P. Étude comparative de la distribution florale dans une portion des Alpes et des Jura. Bull Soc Vaudoise Sci Nat 37, 547–79 (1901).

[56] Aimi T., Matsuda K. & Yuzaki M. C1ql1-Bai3 signaling is necessary for climbing fiber synapse formation in mature Purkinje cells in coordination with neuronal activity. Molecular Brain 16, 61 (2023). 10.1186/s13041-023-01048-4 37488606 PMC10367388

[57] Chen F. Role of CTRP14/C1QL1 in motor coordination and learning across the lifespan. Physiology & Behavior 291, 114799 (2025). 10.1016/j.physbeh.2025.114799 39761721 PMC11788040

[58] Che N. Plasma GFAP as a prognostic biomarker of motor subtype in early Parkinson’s disease. npj Parkinson’s Disease 10, 48 (2024). 10.1038/s41531-024-00664-8 PMC10907600 38429295

[59] Kaisho T. BST-1, a surface molecule of bone marrow stromal cell lines that facilitates pre-B-cell growth. Proceedings of the National Academy of Sciences 91, 5325–5329 (1994). 10.1073/pnas.91.12.5325 PMC43987 8202488

[60] Xu H.-L. The impact of BST1 rs4698412 variant on Parkinson’s disease progression in a longitudinal study. Frontiers in Aging Neuroscience Volume 17 - 2025 (2025). 10.3389/fnagi.2025.1570347 PMC12040838 40308892

[61] De Mori R. Joubert syndrome-derived induced pluripotent stem cells show altered neuronal differentiation in vitro. Cell and Tissue Research 396, 255–267 (2024). 10.1007/s00441-024-03876-9 38502237 PMC11055696

[62] Adams L., Song M. K., Yuen S., Tanaka Y. & Kim Y.-S. A single-nuclei paired multiomic analysis of the human midbrain reveals age- and Parkinson’s disease–associated glial changes. Nature Aging 4, 364–378 (2024). 10.1038/s43587-024-00583-6 38491288 PMC11361719

[63] Zhang W. Neuroprotective effect of a medium-chain triglyceride ketogenic diet on MPTP-induced Parkinson’s disease mice: a combination of transcriptomics and metabolomics in the substantia nigra and fecal microbiome. Cell Death Discovery 9, 251 (2023). 10.1038/s41420-023-01549-0 37460539 PMC10352270

[64] Chew G. Transcriptomic imputation of genetic risk variants uncovers novel whole-blood biomarkers of Parkinson’s disease. npj Parkinson’s Disease 10, 99 (2024). 10.1038/s41531-024-00698-y PMC11078960 38719867

[65] McEwan David G. PLEKHM1 Regulates Autophagosome-Lysosome Fusion through HOPS Complex and LC3/GABARAP Proteins. Molecular Cell 57, 39–54 (2015). 10.1016/j.molcel.2014.11.006 25498145

[66] Gokuladhas S., Miller C., Cooper A. A. & O’Sullivan J. M. Inherited Genetic Variation in Parkinson’s Disease: Convergence on Impaired Autophagosome-Lysosome Fusion Through the Altered Expression of mRNA Isoforms. Molecular Neurobiology (2025). 10.1007/s12035-025-05101-2 PMC12433354 40457026

[67] Liang C. Mitochondrial microproteins link metabolic cues to respiratory chain biogenesis. Cell Reports 40 (2022). 10.1016/j.celrep.2022.111204 35977508

[68] Tabar V. Phase I trial of hES cell-derived dopaminergic neurons for Parkinson’s disease. Nature 641, 978–983 (2025). 10.1038/s41586-025-08845-y 40240592 PMC12095069

[69] Buchman A. S. Parkinson’s Disease and Other Alzheimer’s Disease and Related Dementia Pathologies and the Progression of Parkinsonism in Older Adults. J Alzheimers Dis 100, S197–S209 (2024). 10.3233/JAD-240593 39121125 PMC11619772

[70] Albers M. W. At the interface of sensory and motor dysfunctions and Alzheimer’s disease. Alzheimers Dement 11, 70–98 (2015). 10.1016/j.jalz.2014.04.514 25022540 PMC4287457

[71] Bennett D. A., Shannon K. M., Beckett L. A., Goetz C. G. & Wilson R. S. Metric properties of nurses’ ratings of parkinsonian signs with a modified Unified Parkinson’s Disease Rating Scale. Neurology 49, 1580–1587 (1997). 9409350 10.1212/wnl.49.6.1580

[72] Buchman A. S. Parkinsonism in Older Adults and Its Association With Adverse Health Outcomes and Neuropathology. J Gerontol A Biol Sci Med Sci 71, 549–556 (2016). 10.1093/gerona/glv153 26362440 PMC5014188

[73] Szklarczyk D. STRING v11: protein–protein association networks with increased coverage, supporting functional discovery in genome-wide experimental datasets. Nucleic Acids Research 47, D607–D613 (2019). 10.1093/nar/gky1131 30476243 PMC6323986

[74] García-Marín L. M. Shared molecular genetic factors influence subcortical brain morphometry and Parkinson’s disease risk. npj Parkinson’s Disease 9, 73 (2023). 10.1038/s41531-023-00515-y PMC10172359 37164954

[75] Wong T.-S. G protein-coupled receptors in neurodegenerative diseases and psychiatric disorders. Signal Transduction and Targeted Therapy 8, 177 (2023). 10.1038/s41392-023-01427-2 37137892 PMC10154768

[76] Reilmann R. in International Review of Neurobiology Vol. 149 (eds Stamelou Maria & glinger Günter U. H) 299–306 (Academic Press, 2019). 31779817 10.1016/bs.irn.2019.10.006

[77] Contaldi E., Magistrelli L. & Comi C. T Lymphocytes in Parkinson’s Disease. Journal of Parkinson’s Disease 12, S65–S74 (2022). 10.3233/JPD-223152 PMC9535550 35253782

[78] Karampetsou M., Vekrellis K. & Melachroinou K. The promise of the TGF-β superfamily as a therapeutic target for Parkinson’s disease. Neurobiology of Disease 171, 105805 (2022). 10.1016/j.nbd.2022.105805 35764291

[79] He J., Zhong W., Zhang M., Zhang R. & Hu W. P38 mitogen-activated protein kinase and Parkinson’s disease. 9, 147–153 (2018). doi:10.1515/tnsci-2018-0022 PMC6234472 30473884

[80] Diaz-Ortiz M. E. GPNMB confers risk for Parkinson’s disease through interaction with α-synuclein. Science 377, eabk0637 10.1126/science.abk0637 PMC9870036 35981040

[81] Reich N. & Hölscher C. Cholecystokinin (CCK): a neuromodulator with therapeutic potential in Alzheimer’s and Parkinson’s disease. Frontiers in Neuroendocrinology 73, 101122 (2024). 10.1016/j.yfrne.2024.101122 38346453

[82] Moon H. E. & Paek S. H. Mitochondrial Dysfunction in Parkinson’s Disease. Exp Neurobiol 24, 103–116 (2015). 10.5607/en.2015.24.2.103 26113789 PMC4479806

[83] Ng X. Y. & Cao M. Dysfunction of synaptic endocytic trafficking in Parkinson’s disease. Neural Regeneration Research 19 (2024). 10.4103/NRR.NRR-D-23-01624 PMC11168511 38595283

[84] Delaville C., De Deurwaerdère P. & Benazzouz A. Noradrenaline and Parkinson’s Disease. Frontiers in Systems Neuroscience volume 5 - 2011 (2011). 10.3389/fnsys.2011.00031 PMC3103977 21647359

[85] Lee A. J. Multi-region brain transcriptomes uncover two subtypes of aging individuals with differences in Alzheimer risk and the impact of APOEε4&lt;/i&gt. bioRxiv, 2023.2001.2025.524961 (2023). <http://europepmc.org/abstract/MED/36747803 10.1101/2023.01.25.524961>. PMC13486859 42620705

[86] Wingo A. P. Shared proteomic effects of cerebral atherosclerosis and Alzheimer s disease on the human brain. Nature Neuroscience 23, 696–700 (2020). 10.1038/s41593-020-0635-5 32424284 PMC7269838

[87] Reynolds R. H. Moving beyond neurons: the role of cell type-specific gene regulation in Parkinson’s disease heritability. npj Parkinson’s Disease 5, 6 (2019). 10.1038/s41531-019-0076-6 PMC6470136 31016231

[88] Liu Y. ACAT: A Fast and Powerful p Value Combination Method for Rare-Variant Analysis in Sequencing Studies. The American Journal of Human Genetics 104, 410–421 (2019). 10.1016/j.ajhg.2019.01.002 30849328 PMC6407498

[89] Stegle O., Parts L., Piipari M., Winn J. & Durbin R. Using probabilistic estimation of expression residuals (PEER) to obtain increased power and interpretability of gene expression analyses. Nature Protocols 7, 500–507 (2012). 10.1038/nprot.2011.457 22343431 PMC3398141

[90] Barbeira A. N. Exploring the phenotypic consequences of tissue specific gene expression variation inferred from GWAS summary statistics. Nature Communications 9, 1825 (2018). 10.1038/s41467-018-03621-1 PMC5940825 29739930

[91] Dai Q. OTTERS: a powerful TWAS framework leveraging summary-level reference data. Nature Communications 14, 1271 (2023). 10.1038/s41467-023-36862-w PMC9992663 36882394

[92] Sudlow C. UK Biobank: An Open Access Resource for Identifying the Causes of a Wide Range of Complex Diseases of Middle and Old Age. PLOS Medicine 12, e1001779 (2015). 10.1371/journal.pmed.1001779 25826379 PMC4380465

[93] Szklarczyk D. The STRING database in 2021: customizable protein–protein networks, and functional characterization of user-uploaded gene/measurement sets. Nucleic Acids Research 49, D605–D612 (2021). 10.1093/nar/gkaa1074 33237311 PMC7779004

[94] Pastrello C., Kotlyar M., Abovsky M., Lu R. & Jurisica I. PathDIP 5: improving coverage and making enrichment analysis more biologically meaningful. Nucleic Acids Research 52, D663–D671 (2024). 10.1093/nar/gkad1027 37994706 PMC10767947

[95] Thomas P. D. PANTHER: Making genome-scale phylogenetics accessible to all. Protein Science 31, 8–22 (2022). 10.1002/pro.4218 34717010 PMC8740835

[96] Wishart D. S. PathBank: a comprehensive pathway database for model organisms. Nucleic Acids Research 48, D470–D478 (2020). 10.1093/nar/gkz861 31602464 PMC6943071

[97] Gillespie M. The reactome pathway knowledgebase 2022. Nucleic Acids Research 50, D687–D692 (2022). 10.1093/nar/gkab1028 34788843 PMC8689983

